# The Risk of Persistent Hypertension and Chronic Kidney Disease in Early- and Late-Onset Preeclampsia: A Report From Developing Country

**DOI:** 10.7759/cureus.50488

**Published:** 2023-12-13

**Authors:** Ernawati Ernawati, Aditiawarman Aditiawarman, Agus Sulistyono, Kamalia Hasanah, Salsabilah N Ridfah, M Ilham A Akbar, Erry Gumilar Dachlan

**Affiliations:** 1 Obstetrics and Gynaecology, Universitas Airlangga, Surabaya, IDN

**Keywords:** long term effect, persistent hypertension, late-onset preeclampsia, early-onset preeclampsia, chronic kidney disease

## Abstract

Background and objective: Preeclampsia (PE) has been disproportionately prevalent in developing countries and constitutes a leading cause of maternal mortality, and also has long-term impacts, including renal consequences. This study aimed to explore the risk of persistent hypertension and kidney failure in early-onset PE (EOP) and late-onset PE (LOP) in the five years after delivery.

Methods: This retrospective cohort study included women with a prior history of severe PE or normotensive pregnancy admitted to tertiary hospitals in Indonesia. The blood pressure, body mass index (BMI), urea, creatinine serum, and protein urine were analyzed, and the risk of chronic kidney disease (CKD) after five years was performed using the Kidney Disease Improvement Global Outcomes (KDIGO) classification.

Results: Twenty-seven EOP, 35 LOP, and 30 normotensive cases were included. Mean blood pressure after five years was recorded as 115.6 ± 14.25 mmHg in the normotensive group, 131.82 ± 19.34 mmHg in the LOP group, and 154.96 ± 23.48 mmHg in the EOP group. According to the KDIGO classification, the normotensive group had an average 10% risk of CKD, but severe PE had a risk of CKD greater than 90%. In the severe PE group, the risk of CKD was 20.94 times higher compared to normotensive women (OR 20.94; 95% CI 2.67-163.72, p = 0.004). The risk of CKD in the EOP group was 6.75 times higher than in the LOP group (OR 6.75; 95% CI 2.19-20.76, p = 0.001), whereas persistent hypertension in the EOP group was 5.78 times higher than in the LOP group (OR 5.78; 95% CI 1.91-17.395, p = 0.002).

Conclusions: PE women have a higher risk of CKD than normotensive women. Women with a history of EOP are more likely to develop persistent hypertension and CKD than women with a prior LOP history.

## Introduction

Preeclampsia (PE) is a multisystem disorder that causes high blood pressure and often comes with new organ dysfunction or proteinuria. It is linked to a number of issues that put women and their unborn babies at a higher risk for more complications and problems that will last a lifetime [1]. PE has been significantly prevalent in developing countries and constitutes a leading cause of maternal mortality in low-income countries [2]. Women with PE in developing countries have a higher risk of mortality than those in developed countries, and hypertension is one of the leading causes of maternal mortality in PE [3]. Currently, PE is generally classified into early-onset PE (EOP) and late-onset PE (LOP), which exhibit different clinical manifestations and pathogenesis. EOP is associated with placental insufficiency and defective vascular remodeling, whereas LOP is most likely caused by maternal factors, particularly vascular maladaptation [4,5].

PE has been linked to long-term damage to the kidneys, such as a higher risk of albuminuria [6], chronic kidney disease (CKD) [7], and end-stage kidney disease (ESKD) [8]. Some studies report that kidney dysfunction can resolve in most women with a history of PE [9,10]. However, some women with PE may experience persistently decreased glomerular filtration rate (GFR) and/or proteinuria and/or an increased risk of CKD [11,12]. Only a few data points were presented about its long-term effects on kidney function later in life, mostly in EOP and LOP. EOP and LOP are linked to various outcomes, biochemical markers, and clinical features in both the mother and the fetus. Because of this, it is believed that EOP is a major risk for both the mother and the fetus, while LOP may have milder symptoms [13]. It has been suggested that EOP and LOP have different risks of developing renal impairment in women with PE. Unfortunately, to our knowledge, there is no data or international publication on the risk of CKD following PE in a developing country. Thus, this study intends to explore the risk of renal failure in EOP and LOP five years after PE in Indonesia, one of low middle income country in Southeast Asia. This article was previously posted to the Research Square preprint server on January 23, 2023.

## Materials and methods

This was a retrospective cohort study of women who had previously been diagnosed with severe PE or eclampsia and delivered at Dr. Soetomo General Academic Hospital, one of the largest tertiary referral hospitals in East Indonesia, between January 2013 and June 2014. Using the medical records, we identified all patients who met our criteria and included them in the exposed group, whereas women who recorded having an uncomplicated pregnancy constituted the control group. All exposed cases who lived in Surabaya and were willing to engage in this study were then enrolled. However, women with pre-existing comorbidities such as chronic hypertension, kidney disease, diabetes, autoimmune disease, and cardiac disease at the time of their pregnancy, women who had twins or multiple fetuses, and patients who died over the course of this study were excluded. All samples were contacted and/or visited at their residences before being invited to the hospital for a medical examination and blood sampling test.

PE or eclampsia was defined according to revised International Society for the Study of Hypertension in Pregnancy (ISSHP) criteria: the presence of hypertension (systolic blood pressure >140mmHg and/or diastolic blood pressure >90mmHg), which developed after 20 weeks of pregnancy, and the coexistence of one or more of the following new-onset conditions: proteinuria (spot urine protein/creatinine ratio 430mg/mmol or 4,300 mg protein/24 h or at least ‘‘2+’’ on dipstick testing), and/or other maternal organ dysfunction and/or suspected intrauterine growth restriction (IUGR). Depending on time, EOP was defined as PE that develops before 34 weeks of gestation, whereas LOP was defined as PE that develops at or after 34 weeks of gestation [14]. An uncomplicated pregnancy history was defined as a woman who gave birth between 37 and 42 weeks of gestation, had normal blood pressure, and was without IUGR.

Patients who met the inclusion criteria and did not meet the inclusion criteria were contacted by telephone or made home visits, and subjects willing to participate in the study were asked to come to the hospital to have their blood pressure and kidney function checked. Blood pressure checks are carried out in the hospital using an electronic blood pressure monitor after the patient has rested for 30 minutes.

The primary outcome of the study was the risk of CKD defined according to Kidney Disease Improvement Global Outcomes (KDIGO) 2012 definitions for the classification of CKD based on renal function measured by GFR. Renal function was measured using serum creatinine (Cr) values determined by the Jaffe method and calibrated using isotope dilution mass spectrometry (IDMS) method. Estimated Glomerular Filtration Rate (eGFR) was calculated using the modified Cockcroft and Gault: GFR = (((l40 - age (year)) x weight (kg))/(72 x serum creatinine (mg/dL))) x 0.85. Proteinuria was measured using urinalysis examination. According to these criteria, risk of CKD classified into low risk if the eGFR >60 mL/min/1.73m^2^ and proteinuria <30 mg/g, whereas high risk if the eGFR 30-44 mL/min/1.73m^2^ and proteinuria <30mg/g or the eGFR >60 mL/min/1.73m^2^ and proteinuria >300 mg/g [15].

Basic characteristics data were presented for women with priorly diagnosed EOP or LOP compared to those healthy pregnant women. Categorical data was presented as frequencies (percentage), while continuous variables was presented either as mean (standard deviation/SD) or median (interquartile range/IQR). Differences were analyzed using Kruskal Wallis and the Fisher exact test was applied as an alternative test. Differences in values between groups considered statistically significant if the P-value was <0.05. Odds ratios for the primary outcomes were calculated using logistic regression with 95% confidence intervals (CI). All statistical analysis were performed using SPSS 21 software (IBM Corp., Armonk, NY). The Human Research and Ethics Committee for Basic Science and Clinical Research of Dr. Soetomo General Academic Hospital approved the research protocol (Ref. No: 0842/KEPK/XII/2018). Informed consent was obtained from all participants before the initiation of the study.

## Results

**Figure 1:**
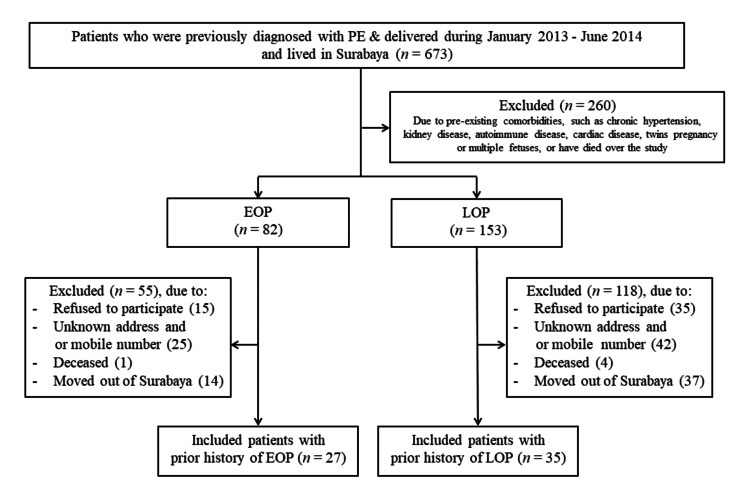
Study flowchart. PE, pre-eclampsia; EOP, early-onset pre-eclampsia; LOP, late-onset pre-eclampsia.

During periods of study, 673 women who were previously diagnosed with PE between January 2013 and June 2014 were listed in their medical records. At the beginning, all patients were assessed for eligibility criteria, of whom 235 met inclusion and exclusion criteria and were enrolled in this study. After excluding some patients due to some reasons, we finally included 62 exposed women with prior PE history, consisting of 27 with EOP and 35 with LOP. We also recruited 30 healthy pregnant women to participate in this study as a control group. Details regarding the study selection are documented in Figure 1.

The basic characteristics and laboratory outcomes of the included participants when first diagnosed with PE are presented in Table 1. Our study found that women with prior PE tend to have a higher mean maternal age compared to the control group with normotensive blood pressure at the time of delivery. The LOP group was dominated by multiparity, whereas the EOP group was dominated by nulliparity. Our results also showed that women with EOP had a significantly higher mean body mass index (BMI) compared to women with LOP and the control group (p = 0.019) and were more likely to have chronic hypertension, kidney disease, and diabetes. However, those with chronic hypertension or CKD at baseline were excluded from further analysis. As expected, the mean systolic blood pressure of women with prior EOP and LOP was significantly higher compared to the control group (p = 0.001), as well as diastolic blood pressure (p = 0.001). Our study also found that gestational age at delivery significantly differed between groups (p = 0.001). Moreover, there was no statistically significant difference in eGFR between the EOP, LOP, and control groups (p = 0.577).

**Table 1:** Baseline characteristics of the sample at delivery *P-value from Kruskal-Wallis test for three groups, while Fisher exact test for each two groups; C, control group; EOP, early-onset severe preeclampsia; LOP, late-onset severe preeclampsia; SD, standard deviation; BMI, body mass index; BUN, blood urea nitrogen; Cr, serum creatinine; eGFR, estimated glomerular filtration rate.

Characteristics	Control (n = 30)	Pre-eclampsia		P-value		
		LOP (n = 35)	EOP (n = 27)	C -LOP	C-EOP	LOP-EOP
Age, mean (SD), years	30.1 ± 6.06	34.46 ± 7.38	33.07 ± 7.01	0.058		
Parity, n (%)						
Nullipara	11 (36.6)	14 (40)	20 (74.1)	0.785	0.005*	0.006*
Multipara	19 (63.4)	21 (60)	7 (25.9)			
BMI, mean (SD), (kg/m^2^)	28.27 ± 4.07	27.07 ± 4.51	30.23 ± 4.94	0.019*		
Gestational age at delivery, mean (SD), (weeks)	38.89 ± 1.38	37.20 ± 2.86	33.3 ± 3.01	0.001*		
Blood pressure at admission mean (SD), (mmHg)						
Systolic	111.66 ± 12.05	160.34 ± 18.22	168.88 ± 15.63	0.001*		
Diastolic	70.66 ± 9.07	102.11 ± 8.20	90.00 ± 16.40	0.001*		
Renal function test at admission						
BUN, n (%) BUN > 21 mg/dL	0 (0)	2 (5.7)	5 (18.5)	0.187	0.014*	1.117
Serum creatinine, n (%) Cr > 1.1 mg/dL	1 (3.3)	0 (0)	2 (7.4)	0.280	0.495	0.104
eGFR, mean (SD) (ml/min/1.73m^2^)	194.93 ± 33.03	171.37 ± 50.86	163.79 ± 57.78	0.577		
Proteinuria (+), n (%)	0 (0)	35 (100)	27 (100)	0.001*	0.001*	

After five years from being first diagnosed with severe PE, the mean systolic and diastolic blood pressure showed significantly higher result in EOP group compared to all groups (p = 0.001). We obtained those women with EOP also had higher mean of BMI (30.23 ± 4.94 kg/m^2^) compared to LOP and control group (27.07 ± 4.51 kg/m^2^ and 28.27 ± 4.07 kg/m^2^, respectively), although failed to show significant difference (p = 0.084). All parameters in renal function showed statistically significant difference between groups, except for the number of positive proteinuria and abnormal protein-to-creatinine ratio between LOP compared to control group (p > 0.05). Additionally, our analysis obtained those women with prior EOP history showed a significant decrease of eGFR compared to all groups (p = 0.001), indicating that the group with prior history of EOP may pose worse renal outcome compared to other groups. Detailed information regarding other characteristics is documented in Table 2.

**Table 2:** Characteristics of the sample five years after delivery *P-value from Kruskal-Wallis test for three groups, while Fisher exact test for each two groups; C, control group; EOP, early-onset severe preeclampsia; LOP, late-onset severe preeclampsia; SD, standard deviation; BMI, body mass index; BUN, blood urea nitrogen; Cr, serum creatinine; eGFR, estimated glomerular filtration rate.

Characteristics	Control (n = 30)	Pre-eclampsia		P-value		
		LOP (n = 35)	EOP (n = 27)	C- LOP	C- EOP	LOP EOP
BMI, mean (SD), (kg/m^2^)	28.27 ± 4.07	27.07 ± 4.51	30.23 ± 4.94	0.084		
Blood pressure at admission mean (SD), (mmHg)						
Systolic	115.6 ± 14.25	131.82 ± 19.34	154.96 ± 23.48	0.001*		
Diastolic	66.53 ± 11.41	81.74 ± 14.49	96.00 ± 16.16	0.001*		
Persistent hypertension, n (%)	0 (0)	9 (25.7)	18 (66.7)	0.003*	0.001*	0.001*
Renal function test, mean (SD)						
BUN, n (%) BUN > 21 mg/dL	0 (0)	0 (0)	10 (37.1)		0.001*	0.001*
Serum creatinine, n (%) Cr > 1.1 mg/dL	0 (0)	0 (0)	10 (37.1)		0.001*	0.001*
eGFR, mean (SD) (ml/min/1.73m^2^)	143.67 ± 33.77	120.80 ± 42.81	97.22 ± 28.71	0.001*		
Proteinuria (+), n (%)	4 (13.3)	11 (31.4)	18 (66.7)	0.087	0.001*	0.006*
Abnormal albumine-to-cratinine ratio, n (%)	4 (13.3)	13 (37.1)	19 (70.4)	0.031*	0.001*	0.010*
Abnormal protein-to-creatinine ratio, n (%)	6 (20)	9 (25.7)	18 (66.7)	0.589	0.001*	0.001*
Persistent proteinuria, n (%)	0 (0)	11 (31.4)	18 (66.7)	0.001*	0.001*	0.006*

Further analysis regarding the incidence of persistent hypertension among women with prior history of PE were varied from 66.7% and 25.7% in EOP and LOP groups respectively. According to the logistic regression analysis, RR of developing persistent hypertension is significantly higher among women with prior history of EOP (RR 5.778; P-value = 0.002; 95% CI 1.919-17.395), as well as the risk of developing CKD (RR 6.75; P-value = 0.001; 95% CI 2.194-20.764) compared to women with prior history of LOP (Table 3). Likewise, according to KDIQO 2012 classification, women with prior severe PE history had significantly higher risk of further developing CKD (RR 20.94; P-value = 0.004; 95% CI 2.679-163.723) compared to normotensive control group.

**Table 3:** The association between the type of PE with the incidence of persistent hypertension and the risk of developing CKD 5 years after diagnosis. *P-value from Kruskal-Wallis test for three groups; PE, pre-eclampsia; EOP, early-onset pre-eclampsia; LOP, late-onset pre-eclampsia; CKD, chronic kidney disease; RR, relative risk; CI, confidence interval.

Variables	Type of PE		Total	P-value	RR (95%CI)
	LOP	EOP			
Persistent Hypertension					
Yes	9 (25.7%)	18 (66.7%)	27	0.002*	5.778 (1.919 – 17.395)
No	26 (74.3%)	9 (33.3%)	35		
Risk of CKD					
High	8 (22.9%)	18 (66.7%)	26	0.001*	6.75 (2.194 – 20.764)
Low	27(77.1%)	9 (33.3%)	36		

## Discussion

This study revealed that five years after delivery, women with a history of PE were at risk for persistent hypertension. EOP and LOP cases had higher blood pressure than normal pregnant women. Results from prior studies indicated that roughly 20% and 8% of women with a history of PE still had hypertension and proteinuria six months postpartum, respectively [16]. The study by Berks et al. showed 39% and 14% of women with a prior history of PE remained with high blood pressure and proteinuria three months postpartum, while 18% and 8% remained with hypertension and proteinuria two years afterward [17].

Our study found that women with EOP have higher blood pressure than LOP. Women with EOP have a risk of developing persistent hypertension 5.7 times higher than LOP. A study by Veerbeek et al. observed that nearly half of women with a history of EOP developed persistent postpartum hypertension. Moreover, the blood pressure of women with an EOP history and pregnancy-induced hypertension was considerably greater than that of women with a LOP history [4]. Comparing EOP and LOP, the maternal vascular response and remodeling pattern revealed distinct vascular adaptations. Therefore, increased vascular resistance can contribute to systolic and diastolic dysfunction and could be a driving force behind the development of chronic hypertension in women with EOP [5]. Consequently, our findings support the idea that cardiovascular risk during pregnancy is predictive of cardiovascular risk later in life, particularly the risk of persistent hypertension [18,19].

Our study indicated that women with a history of severe PE had a greater risk of CKD than the normotensive group. The risk of developing CKD at five years after delivery in severe PE patients is 20 times higher than in normal pregnancies. Patients with a previous history of PE presented lower eGFR and had more cases of persistent protein urine than normal pregnant women. Moreover, eGFR was the lowest in the EOP group. A previous report, including a large cohort study, found hypertensive disorders of pregnancy are associated with an increased risk of subsequent CKD. Renal impairment was also found earlier in women with GH and PE than in normotensive women [10]. Another study also reported a close association between PE and gestational hypertension with the risk of renal disorder in the future [7,20-22].

The risk of CKD in EOP and LOP differed significantly in this study. EOP had a higher risk of developing CKD than LOP. The large cohort study by Vikse et al., with a sample of 570,433 women, showed that PE was a risk factor for the development of end-stage renal disease (ESRD). The risk is higher in PE patients who give birth to premature babies or children with low birth weight, which indicates EOP cases [20]. Irreversible vascular damage due to more severe endothelial damage and inflammatory stress in EOP than in LOP cannot be disregarded [23]. Renal histology of postpartum biopsies on PE patients showed glomerular endotheliosis and vascular injury as classic pathologies features [24] support this finding. PE is suggested to develop kidney disease by causing acute renal impairment, endothelial damage, and podocyte loss [24].

Endothelial dysfunction induced by PE persists after PE in many patients [25]. The remaining endothelial dysfunction is unknown; It could be assumed that endothelial cell disturbance enhances by a high level of Soluble vascular endothelial growth factor receptor-1 (sFlt-1) in women with a history of PE and also due to epigenetic changes induced by PE [26].

Increased levels of sFlt-1 have been found in formerly preeclamptic women [27,28]. This persistence of elevated levels of sFlt-1 in women with a history of PE is expected due to an extra-placental source such as endothelial cells and monocytes. This increased sFlt-1 may lead to changes in the vascular endothelium, increasing the risk of renovascular diseases in later life. Interestingly, increasing sFlt-1 was also found in CKD patients without a history of PE, which positively correlates with proteinuria [29].

 This fact follows EOP. The combination of insufficient immune tolerance to the fetus and poor placentation resulted in the elevation of serum sFlt-1 and decreasing of Placental growth factor (PlGF) level, thus causing vascular endothelial dysfunction, which led to PE manifestation by 34 weeks gestation [30]. It may explain that the risk of developing CKD was higher in EOP than in LOP.

The strengths of this study are mostly related to our hospital (Dr. Soetomo General Academic Hospital), a level 3 and top referral center hospital in eastern Indonesia. At level 3, we have many cases of PE, and almost all cases of EOP and all conservative management are referred to our hospital. Therefore, we have a large number of EOP and LOP cases.

As the limitation of the study, we have to consider that this was a retrospective cohort study. Thus, some information may be missed, like no assessment of renal anatomic abnormalities before pregnancy or when the patient was diagnosed with severe PE, as it is not a standard procedure for initial examination before pregnancy or at the time of diagnosis in our hospital. The high mobility of the population makes this research even more challenging. Since most of the patients in this study were seasonal residents who moved frequently, it was difficult to ascertain their whereabouts, reducing the number of participating patients. However, these limitations do not invalidate our conclusion that EOP is associated with a higher risk of CKD than LOP.

Awareness that severe PE may be associated with a higher risk of persistent hypertension and CKD means that PE patients should follow up regarding this risk. It is necessary to regularly evaluate blood pressure and kidney function to assess the possibility of CKD.

## Conclusions

In women with a history of PE, the outcome of persistent hypertension and/or proteinuria may have renal repercussions. It was found that both systolic and diastolic blood pressures were strongly linked to a history of PE. This has an impact on future kidney problems in both the EOP and LOP groups. In a five-year follow-up, women with severe PE had a higher risk of developing CKD than normotensive women. In addition, women with a history of EOP are more likely to develop persistent hypertension and CKD than women with a prior LOP history.
